# Root Filling

**Published:** 1874-05

**Authors:** C. W. Spalding


### ARTICLE VI.

### *Root Filling.*

BY C. W. SPALDING, D. D. S.

I commenced the filling of root canals some time in the year 1849. I had read what had been previously published upon the subject, and owing to the large field of conservative surgery which this new method promised to open, I felt a strong desire to acquaint myself with the details of the operation as then practiced by the few who had attempted it up to this date.

About this time Dr. E. I. Dunning, of New York, described to me the mode of filling these canals practiced by himself and also allowed me to examine the instruments used by him. His method, as I now remember it, consisted in cutting a narrow fold of gold foil into small pieces and carrying them one by one into the canal before the end of a suitable instrument. Providing myself with a supply of slender instruments, similar to those used by Dr. D., I returned to Saint Louis and entered upon the work with much zeal. My operations were frequent and usually successful.

A year or two afterwards, I think in the summer of 1851, the method employed by Dr. F. K. Badger, then of New Orleans, came to my knowledge. This method has already been described on page 19 of current volume of this journal, and, it appears, was first published in 1854. I immediately commenced a series of experiments with a view of ascertaining what method would best succeed in my hands. My first attempts at preparing the compact cones, used by Dr. Badger, was not very successful, yet I did not abandon it. One day, after having rolled a small piece of foil around a small broach, as directed by Dr. B., it occurred to me that it was then in the best possible position to be carried into the root-canal. I accordingly mounted a number of annealed broaches in this manner, and my next operation was performed with much greater facility and satisfaction than any previous one had been. At once I adopted this modification of Dr. Badger's mode. The broaches employed for this purpose must be re-polished or rubbed smooth after annealing, to allow the pointed roll of foil to slide off easily when the broach is withdrawn. If the point of the broach is too sharp it should be clipped and the foil should be closed over it in rolling. At the point the gold may be easily condensed by rotating it against the thumb nail.

In commencing this operation, select a broach of proper size, armed with a mounting of gold of sufficient diameter to fill the extremity of the canal, or as nearly so as possible, and carry this into the canal of the foramen. The broach must now be slowly rotated in the opposite direction, so as to slightly unwind the foil that is in immediate contact with it. It may now be withdrawn, leaving the gold in place. Condense with probe of whalebone or hickory, and proceed to insert another cone of gold by the side of the first, and so on until the canal is filled. The whole may then be driven forward into the canal either by hand pressure or the mallet —I prefer the latter. After trying different weights of foil I gave preference to No. 4. Cutting the sheet into three or four strips I folded one of these upon itself, making a ribbon say three-tenths of an inch in width. This fold or ribbon was now cut into sections of the length required for the case in hand, and these again divided diagonally, leaving the pieces triangular in shape and of a width and thickness varying with the demands of the case.

In exceptional cases, it is no doubt well to enlarge the canal before filling, but except where the conditions are favorable the liability of forming a shoulder against which the filling is sure to lodge limits this operation to such canals as are straight and of easy access. The orifice of a canal may, however, be usually enlarged with advantage, but the instrument employed for the purpose should terminate in a point.

Canals of too small a calibre to be filled in the manner described above I have usually filled with gold wire. For this purpose I prefer a rather stiff wire, and have found the alloyed gold used in spiral springs to possess the requisite properties. The wire is first reduced to fill the canal as accurately as possible. It is then cut nearly off, of a length that will allow it to project somewhat into the pulp chamber. A little motion will then sever the slight connection remaining, when the plug may be firmly driven by other means. This wire must not be reduced to a point, for in case it reaches the extremity of the root it must be too large to pass beyond the foramen.

I am doubtful of the necessity for filling *very* small canals at all; but it is so quickly done in the manner I have just described that I have always preferred in this class of operations to leave nothing undone that might contribute to success.—*Missouri Dental Journal.*

